# What makes up good consultations? A qualitative study of GPs’ discourses

**DOI:** 10.1186/1471-2296-14-62

**Published:** 2013-05-16

**Authors:** Kaatje Van Roy, Stijn Vanheule, Myriam Deveugele

**Affiliations:** 1 Department of Psychoanalysis and Clinical Consulting, Ghent University, H. Dunantlaan 2, 9000 Ghent, Belgium; 2 Department of General Practice and Primary Health Care, Ghent University, UZ Gent 1K3 De Pintelaan 185, Ghent, 9000, Belgium

**Keywords:** Discourse, General practitioner, Qualitative research, Consultation, Belgium

## Abstract

**Background:**

In medical literature, several principles that define ‘good consultations’ have been outlined. These principles tend to be prescriptive in nature, overlooking the complexity of general practitioners (GPs)’ perspectives of everyday practice. Focusing on perspectives might be particularly relevant, since they may affect decisions and actions. Therefore, the present study adopts a bottom-up approach, analyzing GPs’ narratives about ‘good’ and ‘bad’ consultations. We aimed at describing the range of discourses GPs use in relating on their practice.

**Methods:**

Semi-structured interviews were conducted with 19 Belgian GPs. By means of a qualitative analysis, the authors mapped patterns in the interview narratives and described the range of different discourses.

**Results:**

Four discourses were identified: a biomedically-centered discourse, a communication-focused discourse, a problem-solving discourse and a satisfaction-oriented discourse. Each discourse was further specified in terms of predominant themes, problems the GPs prefer to deal with and inherent difficulties. Although most participants used elements from all four discourses, the majority of the GPs relied on an individual set of predominant discourses and focused on a limited number of themes.

**Conclusion:**

This study clearly indicates that there is no uniform way in which GPs perceive clinical practice. Each of the participants used a subtle mix of different criteria to define good and bad medical consultations. Some discourse elements appear to be rooted in medical literature, whereas others are of a more personal nature. By focusing on the limitations of each discourse, this study can shed new light on some of the difficulties GPs encounter in their daily practice: being confronted with specific problems might be an effect of adhering to a specific discourse. The typification of different discourses on consultations may function as a framework to help GPs reflect on how they perceive their practice, and help them manage some of the challenges met in daily practice.

## Background

In medical literature, principles and guidelines that define ‘good medical practice’ or ‘good consultations’ are continually being developed. For instance, literature on evidence-based medicine (e.g. [1-4]), shared decision-making (e.g. [5-7]) and medical competencies (e.g. [8]) is vast in this respect. These principles and guidelines are corroborated by research findings that depict the way medical practice can best take shape, and aim to *prescribe* practitioners’ actions and attitudes. However, such a prescriptive approach is limited since it treats all individuals of a professional group, such as General Practitioners (GPs), as similar in how they make sense of their clinical practice and neglects how individual GPs actually experience their everyday clinical work.

Previous studies indicate that in medical practice clinical decisions are not only based on scientific knowledge; interpretation and ‘tacit knowledge’ also play an important role [9,10]. Moreover, GPs differ in terms of their experience, capacity, personality and personal values [3,4,11,12]. To further explore this subjective component, qualitative approaches that view GPs as “reflexive, meaning-making and intentional actors” (2003: 49) [13] and that identify patterns in the way they think and speak about their daily practice may be useful [10]. In this paper we adopt such qualitative stance, and view GPs as sense-making agents that actively construct their professional realities [14].

Previous research investigating GPs’ perceptions of what they deem ‘effective health care’ [15] indicates that different criteria are used with respect to how clinical practice is evaluated. This might also apply to the way GPs evaluate consultations with patients, i.e., why certain doctor-patient interactions are deemed rewarding or difficult. Rather than merely outlining criteria that are explicitly mentioned by the participants, the present study intends to outline participants’ perspectives, by taking also into account what is implicitly referred to (e.g. by means of striking word choices or contradictions). By analyzing narratives from interview data, the authors map patterns in the way GPs speak about their daily practice. Following a bottom-up approach [16] that uses GPs’ descriptions and concrete examples of good and bad practice, this study examines a) the ideas and concepts used by GPs in relation to their work, b) the themes that spontaneously recur in the context of descriptions of their practice, and c) the difficulties highlighted as obstacles to good practice. Focusing on these aspects, the *discourses* the participating GPs characteristically make use of are mapped out. Discourses are understood as reflecting the angle from which someone constructs reality [17]. Since language is considered crucial in the subjective sense-making process [13,18,19], this study focuses on the language that GPs use to construct narratives about their consultations. For reasons of clarity, the interview data from which the analysis started will be called ‘*narratives*’, whereas the results of the analysis will be denominated ‘*discourses*’.

## Methods

### Data collection and sampling

The first author, a female researcher with a degree in medicine and psychology, conducted semi-structured interviews with 19 Belgian GPs between June 2011 and June 2012. All interviews were audio-recorded. GPs were recruited by means of snowball sampling [20]. Four GPs were contacted by telephone and invited for an interview on the broad topic of ‘consultations with patients.’ At the end of each interview, participants were asked to give the name of one or more colleagues that could be contacted for an interview. It was assumed that this method would facilitate a trustful atmosphere during the interviews. Only one GP declined participation due to time constraints. In order to obtain sufficient variation in the sample, demographic characteristics were taken into account when selecting new participants among the candidates named. All participants gave written and oral informed consent and completed a short questionnaire designed to gather demographic data and information about the GP’s practice.

In order to elicit GPs’ narratives on their practice, it was decided to opt for interview questions that were as open as possible, yet specific enough. Therefore, the semi-structured interview contained the following questions:

1. What do you consider to be a ‘good’ consultation? Describe this in general terms. What are the components of a good consultation according to you? Give one or more examples of a good consultation.

2. What do you consider to be a ‘bad’ consultation? Give examples of what you would consider to be a ‘less good’ or a ‘bad’ consultation.

In between successive interviews, the interview questions were repeatedly evaluated in terms of their appropriateness to provide the kind of data that was aimed at, i.e., rich narratives. Assessed as well suited, the interview questions remained the same during all interviews. In order to elicit rich narrative material special attention was paid to encouraging the participants to speak freely.

Following each interview, the interviewer made reflective notes regarding observations and impressions during the interview. Potential preconceptions due to the interviewer’s background were cut back by reflections and discussions among the researchers on the one hand, and by a constant focus on asking open questions during the interviews on the other hand. When the first nine interviews were complete, an initial stage of saturation was perceived by the authors. The interviews were transcribed verbatim and an in-depth analysis of the data was carried out. This analysis led to the identification of four characteristic discourses. Following this, ten more interviews were carried out with the aim of refining and validating the intermediate findings. Data collection was terminated when saturation was reached (n=19) [21].

This study was approved by the Ghent University Committee for Medical Ethics.

### Participants

Nineteen GPs participated in this study (see Table 1). All participants lived and worked in Flanders, the Dutch-speaking region of Belgium, and had received their medical training at a university in this region. Of the participants, 11 were male and eight female; age ranged between 28–63 years (mean 42.42; SD 10.42). Their years of experience as a GP ranged from one to 39 years (mean 16.84; SD 11.27); seven participants worked in a solo practice, 12 in a group practice.

**Table 1:** Demographic characteristics participants

**GP**	**Gender**	**Age range**	**Years experience as GP**	**Solo vs group**
**GP 1**	M	60-64	37	Solo
**GP 2**	M	40-44	23	Solo
**GP 3**	M	30-34	7	Group
**GP 4**	F	40-44	17	Solo
**GP 5**	M	45-49	20	Solo
**GP 6**	M	35-39	10	Group
**GP 7**	M	60-64	39	Solo (15 yr duo)
**GP 8**	M	45-49	19	Duo
**GP 9**	F	25-29	1	Duo
**GP 10**	F	45-49	23	Duo (14 yr solo)
**GP 11**	M	50-54	26	Group
**GP 12**	F	35-39	11	Solo
**GP 13**	M	50-54	26	Solo
**GP 14**	F	25-29	2	Duo
**GP 15**	F	35-39	10	Group
**GP 16**	M	50-54	27	Group
**GP 17**	F	40-44	13	Group
**GP 18**	M	25-29	2	Group
**GP 19**	F	35-39	7	Group

### Analysis

The data were examined with a focus on the language used by participants during each interview. As stated above, the use of specific language is indicative of the broader *discourse* individuals employ in terms of making sense of (parts of) reality [11]. In line with Parker [22] and Foucault [23], the use of particular discourses can be thought of as “practices that systematically form the objects of which they speak” (1972: 49) [23]. Indeed, according to Crowe [18] “language constructs how we think about and experience ourselves and our relationships with others” (2005: 56). Moreover, specific jargon makes up patterns by means of which the meaning of practices and relationships is understood [19,24,25].

**Figure 1:**
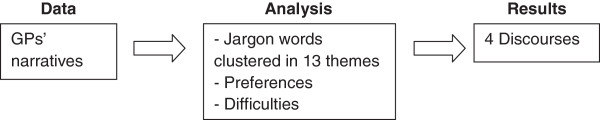
Overview of analytic process.

The method used in this study was guided by the analytical steps outlined by Parker [19,22], which is particularly well suited for finding discursive patterns in narrative data. Firstly, the interview transcripts were analyzed with the aim of identifying the type of language used by the participants in their responses. The language used by participants was then grouped into broader clusters of *jargon* words [19,20]. The interview transcripts were then re-examined to a) gather fragments that reflected the types of clinical problems GPs expressed preference for, and b) the difficulties they encounter in their practice. For the first nine interviews, 12 clusters of jargon words were discerned and grouped into corresponding themes. In the ten subsequent interviews only one additional theme was discerned (see Table 2). Following repeated discussions between the first two authors, 13 clusters of jargon words and their corresponding themes were then grouped into four discourses. The second author is a male university professor in clinical psychology, a psychoanalyst and has experience in doing qualitative research. A brief visual presentation of the analysis is provided in Figure 1.

**Table 2:** Themes arising during first and second phase of analysis

**First phase of analysis**	**Second phase of analysis**
Decoding messages	Time management
Executing guidelines	
Convincing patients	
Advising patients	
Pragmatic solution seeking	
Medical expertise	
Patients’ satisfaction	
Referring patients	
Economic thinking	
Medically interesting cases	
Positive rapport	
Verbalizing intuitions/non-verbal behavior	

Quality control was built into the analyses in the form of discussions between the first and second authors of this study during the whole process. Attention was paid to ensuring that the codes covered all relevant data [26]. Consultations between the first and second author focused on identifying which discourses could be discerned in the initial codes. The final results were verified by the third author, who is a female university professor, a psychologist, experienced in doing qualitative research and responsable for communication skills training at the faculty of medicine. She particularly examined whether the discourses identified were supported by relevant interview fragments [20,26,27].

## Results

### Discourses

A detailed analysis of the GPs’ narratives resulted in the identification of four discourses: a biomedically-centered discourse, a communication-focused discourse, a problem-solving discourse and a satisfaction-oriented discourse, each specified in terms of predominant themes, preferred problems and typical difficulties (see Table 3). These themes and discourses were identified across the interview data as a whole, and thus the description of the four discourses is not a typology of individual GPs. The discourses are illustrated by interview quotes (that were translated from Dutch to English).

**Table 3:** Overview of the four GP discourses on consultation identified

	**Themes**	**Preferred problems**	**Difficulties**
**Biomedically**-**centered discourse**	- Executing guidelines	- Medically ‘interesting’ problems	- Lack of knowledge or expertise
	- Scientific interest		
	- Referring patients to specialists	- Problems that can be framed biomedically	- Making bad impression to specialists
	- Medical expertise		
**Communication**-**focused discourse**	- Decoding messages and signs	- Problems with deeper psychosocial ground	- Not being able to decode messages
	- Verbalizing thoughts and emotions		- Patient not open to communication
**Problem**-**solving discourse**	- Pragmatic solution seeking	- Clear-cut questions or problems for which the GP can provide a satisfying solution	- Stress of finding solutions for problems
	- Advising patients		
	- Convincing patients		- Finding right balance in advising and convincing
	- Time management		
**Satisfaction**-**oriented discourse**	- Satisfying your patients	- Nature of problem of minor importance; satisfaction and patient’s expectations rule	- Angry, dissatisfied, demanding or intimidating patients
	- Economic thinking		
	- Positive rapport		
			- Patient’s lack of trust

### Biomedically-centered discourse

#### General description

In this discourse, the language used by participants largely refers to science, medical knowledge, standards and guidelines, and the organization of medical care. A good GP is depicted as an expert in biomedical science, someone who has extensive technical expertise, knowledge of diseases and/or experience with the organization of the medical world. In this discourse consultations are defined in terms of making and formulating diagnoses and prognoses, applying medical interventions, and taking up a mediating role in relation to specialist care.

#### Themes

GPs that made use of this discourse frequently referred to the *application of medical standards* and favored clear-cut problems that have clear-cut treatment guidelines. For instance, in describing a ‘good’ consultation, GP 2 referred to identifying a biomedical problem (high blood pressure) and his response (i.e., measuring the patient’s blood pressure a second time, making a follow-up appointment, reviewing the patient’s medication). Moreover, an attitude of *scientific curiosity* i.e., the potential discovery or revelation of a rare or unusual diagnosis, was regarded as inherent to a ‘good’ consultation, as illustrated by GP 5: “You also have scientific expectations (…), scientific curiosity: what will emerge from this?”

Some GPs associated ‘good practice’ with the correct *referral* of patients with serious medical problems to specialists. GP 5, for instance, repeatedly brought up the subject of making referrals, e.g., by describing a recent case of a seriously ill woman he had to refer to a specialist, his reaction to a patient’s demand for (an unnecessary) referral, and the importance of having a good relationship with specialists. “I think that being a GP (…) you should be able and dare to urge colleague-specialists [to see a patient], but in such a way that you do this seriously” (GP 5). By frequently commenting on the referral of patients, this GP underlined the inscription of his professional identity in a world of medical experts.

#### Preferred problems

Elements of ‘good’ consultations noted by some GPs included being exposed to medically ‘interesting’ problems and being acknowledged as an expert in biomedical matters. This was illustrated by GP 4 and GP 18, who referred to their prompt recognition of a (benign) medical condition that worried their patients. For example, in response to one patient who was anxious about an unusual rash, GP 4 stated: “And then I started to think, ‘I have an idea about what this is, it probably won’t be bad’ and then he showed me and I said ‘Yes! Look, it’s this, you don’t have worry at all, it appeared just like that and it will disappear in the same way’. And that’s so delightful….”

#### Difficulties

Missing a diagnosis or lacking medical knowledge (e.g. regarding dermatological problems (GP 5) or palliative pain management (GP 4)), technical experience (e.g. surgical (GP 5)), or orthopedic expertise (GP 4, 10) were frequently mentioned as examples of ‘bad consultations’. Other difficulties include making a bad impression on specialists, worrying about minor medical problems, or not being able to correctly assess a situation. Moreover, consultations without ‘interesting’ medical complaints were perceived as tedious by GPs who put a strong focus on medical conditions. In this respect, GP 3 reported experiencing difficulties giving examples of what he considered to be a ‘good consultation’. He stated that at the end of his working day he sometimes doesn’t actually remember the patients that visited him: “Like in any job, there are things that occur ten times per day and which you probably try to do well, but that’s more of a routine, I don’t suppose afterwards you think ‘great’” (GP 3).

### Communication-focused discourse

#### General description

In this discourse, the focus is on the communicative elements of a consultation. ‘Decoding’ the patient’s message or ‘deciphering’ what the patient is consulting for is of major importance. In contrast to the biomedically-centered discourse, clinical signs and symptoms are not considered exclusively in terms of biomedical diseases, but also seen as indicators of psychosocial distress to which the GP should attend. The consultation is perceived as a communicative context in which emotions and opinions should be ‘verbalized’ and attuned. In this discourse, a good GP is described as being able to ‘read between the lines’, or as having an eye for the psychosocial factors that might contribute to the patient’s problem. A good GP should have the skills to communicate his/her intuition and cope with his/her emotions during consultations. Conversely, consultations are described as difficult if the GP’s decoding and communicative effort proves to be in vain.

#### Themes

Some GPs explicitly referred to the *decoding* of patients’ messages, suggesting that one should often look for “the complaint behind the complaint” (GP 1) and listen to “what is not said as well [as what is]” (GP 4). The problem presented might not even be clear to the patient him/herself, as noted by GP 7: “What is most important is that the patient, when he leaves, got what he came for, *consciously or unconsciously*”. Decoding the patient’s message also includes taking into account non-verbal behavior, as noted by GP 7: “I think that a good consultation has to be [one] where the patient can express, verbally or with his attitude, what he came for”. This is inherently linked to an interest in the broader contextual or psychosocial determination of the problem, as illustrated by GP 1: “When you visit an elderly woman, and if it was recently Mother’s Day and she didn’t see anyone [in her family], and the woman is not feeling well, you don’t have to make a big fuss about it or look further, you don’t need to administer tests to deduce that she could be depressed. Just look at the bigger picture”.

Other GPs emphasized that ‘good practice’ requires investment in communication. For some, *verbalizing* emotions or intuitions was mentioned as important. The patient’s verbal and non-verbal behavior is monitored closely and if a problem is perceived, this will be communicated. For example, GP 4 stated: “Sometimes I say, ‘I can see it, you’re not happy, it is as if you want something else. What do you want? What in fact do you want, or what did you expect?’” GP 14 referred to a moment when she had communicated non-verbal signs of disagreement between a man and his wife, stating [to the interviewer]: “You need to pay attention to the signals between people, and I think it was good that I had noticed this”. Several GPs mentioned bringing something up for a second time with a patient if they felt something was not right. GP 1 remarked: “You immediately feel it in the relationship, like, ‘you’re worried about something or I am worried about something’, then you bring that up immediately. ‘I had the feeling that last time we did not really get there, or that I didn’t hear or understand what exactly it was about. I felt troubled’, then I try (…) to talk it through in order to be on the same wavelength again”. Similarly, all of the examples provided by GP 17 came down to the importance of mutual understanding: the need for an open stance with respect to the patient’s frame of reference and the verbalization of possible points of misunderstanding or conflict. By articulating her reluctance to give a certificate to a young patient who claimed to be unable to work, and instead helping the patient verbalize the real reason for the request, GP 17 was able to expose the underlying problem: a lack of knowledge about child-care organizations. “Why was this good? Well, because, in spite of a question that bores me (…), I tried to understand why she thinks she cannot work” (GP 17).

#### Preferred problems

Problems with a psychosocial basis are preferred. They are experienced as challenges that provide work satisfaction. For instance, with reference to the factors contributing to a patient’s somatic complaints (vague gastric complaints), GP 1 asserted: “Well, I think that when you offer a certain interpretation, people can get into an unguarded moment. These are delightful moments, because then they come closer to themselves. It’s nice for yourself as well, because you come closer to a possible solution, but that solution is not for me, they have to find it themselves”. In this discourse, interpersonal and psychosocial problems are experienced as both challenging and stimulating.

#### Difficulties

Difficulties can arise when the GP is unable to accurately decode the message or cues. For example, GP 1 stated: “It was a false feeling of a consultation being good”. This GP stated that, although he had a good rapport with his patient, it took 15 years for the patient to admit to having a severe alcohol problem (which explained many of her persisting complaints). Similarly, with reference to a patient who had lied about his drinking behavior and convinced him to fill out forms, GP 7 described it as: “Being duped (…) being deceived, or not having seen through it”. Some GPs report patients’ ideas on communication or patients’ poor communicative capacities as posing difficulty at times. GP 1, for instance, stated: “But people have to be open to this. Some people are absolutely not into this. If I ask [a patient who consults with a sore throat]: ‘A sore throat? Is everything going ok lately? Are there problems at home or things like that…?’, [some will answer]: ‘I’ve got a sore throat.’ That happens”.

### Problem-solving discourse

#### General description

In this discourse, the focus is on identifying problems and providing solutions. As derived from the Latin verb consulere and consultare, i.e., to apply to someone for advice or information [28], a ‘consultation’ can be defined as a situation where someone (a patient) presents with a problem and hopes to find a solution. The aim of the GP is to solve the problem pragmatically, making use of a broad range of tools. In this discourse, consultations are sometimes described as difficult if the patient’s problems and demands are vague, and if, in relation to these problems, the GP’s toolbox proves insufficient.

#### Themes

Some GPs referred to the idea of being *pragmatic*, aiming to ‘give’ the patient ‘something palpable’ at the end of the consultation. This might consist of a recommendation, a prescription, information, or an opinion about the development of a problem. This was illustrated by GP 2: “Generally, your patient will be satisfied if you can reach an objective, or if you make a concrete plan about how you will try to solve something. I think that’s most important to me” and GP 8: “A consultation, however good or pleasant it may be, is still a functional encounter, it has to yield something”. For GP 8, a consultation must be ‘functional’, in that there has to be a clear before and after; it must achieve a goal. GP 8 also acknowledged that this ‘functionality’ can be broadly interpreted. For instance, reassuring a patient’s wife, letting her voice her frustration about specialists and the changes in the couple’s life due to the diagnosed disease were considered equally as functional as setting up a treatment plan for her husband. Both GP 9 and 18 stressed the importance of structuring consultations and demarcating problems. GP 9 stated: “Firstly, I think there needs to be some structure in the consultation, so that it‘s not skipping from one subject to another”. Commenting on an example of a good consultation, GP 10 stated: “What I considered good in this consultation? I like to manage, I like to structure and organize things”. In this context, three GPs (GP 4, 14, and 18) highlighted the importance of a thorough ‘stock-taking’ of the patient’s questions at the beginning of a consultation.

In the context of structure and management, five GPs (GP 10, 11, 12, 15, and 16) highlighted the importance of ‘*time management*’. GP 15 and 16, for example, regarded (the feeling of) ‘having enough time’ as the first condition for a good consultation and GP 12 mentioned a ‘good flow’ as a crucial aspect of a good consultation. GP 11 highlighted the challenges associated with this ‘time management’ factor and evaluated one particular consultation as ‘good’ because he managed to complete it in good time, even though he had expected it to be difficult.

Some GPs stressed their *advising*-*convincing* role, which can range from responding to a patient’s request for advice to trying to convince the patient that he or she has a particular problem (e.g. smoking behavior), and subsequently providing advice. The type of advice that is given concerns medical matters as well as psychosocial matters (e.g. family problems, financial difficulties or emotional problems). GP 3 illustrated this when describing the content of his job: “Well, finally, just being a scientific advisor, [this is] the most simple [aspect], but indeed apart from that, also giving advice on certain family matters, divorces, deaths, advice on how to cope with emotions, how they [the patients] would literally be better off leaving someone, or not, whether some of their habits are good, and others not”.

#### Preferred problems

In this discourse, patients with clear-cut questions or problems are preferred. Patients with vague demands are often experienced as irritating, as illustrated by GP 3, when talking about a paranoid patient: “It’s a man who doesn’t put his cards on the table (…) he invents all kinds of stories. It’s almost impossible to figure him out, like, what exactly is he looking for?” This contrasts with the communication-focused discourse, where such patients are deemed challenging and interesting.

#### Difficulties

The urge to provide a ‘solution’ to the problems presented can be experienced as stressful by a GP. For example, GP 2 recalled a consultation where he had ‘promised’ a patient that his backache would be better in two weeks, which turned out not to be the case: “Maybe I created false expectations during that first consultation, … but I always try to give something concrete at the end of a consultation, in that I say: ‘I expect this’ and, well, perhaps yesterday I got what was coming to me (laughing)”. Similarly, GP 12 reported the difficulty she experienced when she fruitlessly attempted to solve a couple’s communication problems surrounding the terminal character of the husband’s cancer. In this situation, the position of mediator the GP found herself in seemed impossible to hold.

Several GPs mentioned having difficulty finding the right balance between advising and convincing patients. Too strong a focus on persuasion might induce resistance on the part of the patient. However, refraining from advising a patient is not deemed appropriate either. For example, GP 1 referred to the importance of expressing his personal opinion, especially in relation to complex medical matters. “Not actually deciding for the patient, but daring to offer an opinion, [which is] something I notice to be different with younger physicians, [who say to their patients]: you have the information, the choice is up to you”.

### Satisfaction-oriented discourse

#### General description

In this discourse, the focus is on patient satisfaction and a smooth doctor-patient interaction. Some GPs repeatedly referred to the importance of the patient’s satisfaction, either for internal (such as the GP’s self-esteem) or external reasons (such as economic motives). In the latter case, the patient is understood as a client who consumes the GP’s services. Here, a good GP is defined as having pleased the patient, who will consult again the next time. Affective elements, such as a positive rapport and trust, also play an important role in this discourse.

#### Themes

Evidently, most GPs prefer their patients to be *satisfied* with the consultation, but some GPs’ functioning seems highly dependent on the patient’s satisfaction. This was illustrated by GP 2, who stated: “I am satisfied if I think or feel my patient is satisfied”. When asked to extract the elements that made him evaluate an example as good, GP 13 repeatedly stressed prioritizing the patient’s wishes, e.g., the patient’s wish not to speak about her depression or the patient’s wish to abstain from further medical intervention.

Pleasing the patient was occasionally motivated by *economic factors*. This was illustrated by some GPs’ concern for losing patients (patients consulting another GP). GP 5, for instance, stated that he would rather comply with a patient’s request for a referral than run the risk of the patient consulting another GP for a second opinion. This statement was immediately followed by the reflection that “in these times, we’re all competitors” (GP 5).

Some GPs referred to the importance of a *positive rapport* or connection with the patient during a consultation. GP 8 stated: “A good consultation means a good connection between two people. This means, both parties leaving with a content feeling. I do find this very important”. When reporting an example of a ‘good’ consultation, GP 7 outlined its main determinants, stating: “He [the patient] felt at ease, I felt at ease”. Similarly, GP 6 offered an example of a good consultation, stating: “It was a guy my age, [there was] a connection, in that we are both interested in sports, and this is nice if there is already a connection”. This emphasis on a positive atmosphere can stem from the GP’s personal needs, as illustrated by GP 8 who notes having experienced that, in the long term, “extra input into the affective part of a consultation” does not contribute to a better doctor-patient relationship or better medical outcomes: “The affective part, the mere affective part has diminished [over the years]. Perhaps because I need it less (…). So that extra [affective] input is not profitable. Not for me and not for the patient. Well, that’s only a satisfaction of needs, but it’s not effective, in no way”. This emphasis on positive affective elements of a consultation differs from what was described in the communication-focused discourse, in which communication in relation to a broad range of topics (positive *and* negative) is stressed.

#### Preferred problems

In contrast to the discourses outlined above, in this discourse the type of problem is less important than the match between the GP and patient’s expectations.

#### Difficulties

Angry, dissatisfied, demanding or intimidating patients are experienced as difficult in this discourse. For GP 2, a ‘bad’ consultation was one in which the patient continued to ask for more information, even after he had responded to the patient’s questions for quite a while. A patient’s lack of trust in the GP is also mentioned as problematic. GP 4, for instance, reported experiencing extreme difficulty when a patient expresses distrust for the GP: “A bad consultation is when you feel, ‘oh there is no trust, they doubt you’”. Conversely, GP 19 emphasized the doctor’s need to trust the patient, referring to distrust on the physician’s side when a patient asks for certificates.

### GPs’ preferences in the use of discourse

All four discourses identified in this study were, to a certain extent, used by the majority of the participating GPs. Reporting on their professional experiences, almost all GPs referred to one or more biomedically-centered themes, communication-focused themes, problem-solving themes and satisfaction-oriented themes. However, in most GPs’ narratives, the predominant presence of particular themes and discourses was observed (see Table 4).

**Table 4:** Preferred discourses and themes per participant

**GP**	**Themes**
**GP 1**	Decoding (D2), verbalizing (D2), advising-convincing (D3)
**GP 2**	Guidelines (D1), pragmatic (D3), satisfying patients (D4)
**GP 3**	Guidelines (D1), scientific interest (D1), advising-convincing (D3)
**GP 4**	Medical expertise (D1), decoding (D2), verbalizing (D2), positive rapport (D4)
**GP 5**	Guidelines (D1), scientific interest (D1), satisfying patients (D4), economic thinking (D4)
**GP 6**	Guidelines (D1), medical expertise (D1), decoding (D2), positive rapport (D4)
**GP 7**	Decoding (D2), time management (D3), positive rapport (D4)
**GP 8**	Verbalizing (D2), pragmatic (D3), positive rapport (D4)
**GP 9**	Pragmatic (D3), advising-convincing (D3)
**GP 10**	Decoding (D2), pragmatic (D3), advising-convincing (D3), time management (D3)
**GP 11**	Decoding (D2), pragmatic (D3), time management (D3)
**GP 12**	Scientific interest (D1), pragmatic (D3), time management (D3), satisfying patients (D4)
**GP 13**	Guidelines (D1), satisfying patients (D4)
**GP 14**	Decoding (D2), verbalizing (D2), pragmatic (D3)
**GP 15**	Decoding (D2), time management (D3)
**GP 16**	Medical expertise (D1), decoding (D2), advising-convincing (D3), satisfying patients (D4)
**GP 17**	Decoding (D2), pragmatic (D3)
**GP 18**	Medical expertise (D1), pragmatic (D3)
**GP 19**	Pragmatic (D3), advising-convincing (D3), positive rapport (D4)

D1 = discourse 1 = biomedically-centered discourse; D2= discourse 2 = communication-focused discourse; D3 = discourse 3 = problem-solving discourse; D4 = discourse 4 = satisfaction-oriented discourse.

## Discussion

This study examined GPs’ narratives about what they deem to be ‘good’ or ‘bad’ consultations in their clinical practice. The narratives were found to be patterned in terms of four discourses: a biomedically-centered discourse (with explicit reference to medical guidelines, scientific interest and/or referral to specialists), a communication-focused discourse (which focused on decoding messages and/or verbalizing thoughts and emotions), a problem-solving discourse (referring to the pragmatics of a consultation or on advising or convincing patients) and a satisfaction-oriented discourse (focusing on satisfying patients, either for internal or external reasons, and/or on creating a positive rapport with the patient). Each discourse identified was further specified in terms of preferred problems and inherent difficulties.

The four discourses appear to reflect distinct ways in which GPs approach their clinical practice, decipher the components of good and bad consultations, and qualify what they experience as rewarding or tedious in their practice. This study indicates that there is no uniform way in which GPs perceive clinical practice. Each of the participants appeared to be using a subtle mix of different criteria to define what they deem good and bad medical practice.

The themes and discourses identified appear to be related to distinct sources. On the one hand, the language used in particular discourses, such as the adherence to ‘medical standards’, ‘good communication skills’ or ‘patient satisfaction’, is clearly rooted in medical literature. Similarities with descriptions of medical competencies (such as Canmeds roles [29]) can also be noted. On the other hand, the present study demonstrates that GPs’ narratives are more complex and that personal criteria are also present in GPs’ descriptions of good and bad consultations. For example, some participants defined ‘good consultations’ as those in which the GP stands behind the proposed treatment, where the GP does not succumb to a patient’s demand if it conflicts with medical guidelines, or inversely, where the patient’s perceived wish is prioritized. ‘Good consultations’ were also described as those in which the GP’s professional identity in relation to medical specialists was established; where the consultation was well structured; where a complex situation was dealt with efficiently; where a distinct before and after could be identified; or where there was a warm and trusting interaction between the physician and the patient.

These examples illustrate that, apart from common influences, personal factors also determine GPs’ narratives about their clinical practice. Previous studies have explored subjective factors associated with different aspects of the medical profession. For example, Epstein [9] states that “physician factors such as emotions, bias, prejudice, risk-aversion, tolerance for uncertainty, and personal knowledge of the patient also influence clinical judgment” (1999: 834). By adopting a bottom-up approach, this study aimed at getting a broad and varied picture of the way individual GPs perceive their practice. In line with other authors who stated that GPs’ perceptions “control how they are doing their job” [30], we believe that the elaboration of different discourses might shed light on what drives GPs during their consultations and might help us gain further insight into clinical decision-making processes.

Focusing on discourse can also shed new light on some of the difficulties GPs encounter in their daily practice. As this study demonstrated, each discourse contains certain limitations. For instance, experiencing the urge to provide solutions and thus repeatedly ‘promising’ to cure a patient reflected one of the limitations of the problem-solving discourse; granting a patient’s request to be referred to a specialist while deeming this medically unnecessary reflected one of the limitations of the satisfaction-oriented discourse; and experiencing consultations for ‘ordinary’ medical reasons as tedious reflected one of the limitations of the biomedically-centered discourse. The link between a certain discourse and its inherent difficulties might be particularly relevant, as this study demonstrated that most participants used certain discourses more predominantly than others. Participants may thus be predominantly confronted with those difficulties associated with their preferred discourses. A detailed description of the diversity in GPs’ narratives on consultations might provide an alternative approach to exploring the difficulties associated with implementing good medical practice principles. While previous research has focused on the extraction of distinct factors that are correlated with these difficulties, such as limited awareness of guidelines, lack of time, poor quality of guidelines, patient preferences, and personal and professional experiences [31-34], a qualitative analysis of GPs’ discourses on consultations takes into account what Sweeney [4] identified as the ‘complexity in primary care’. Moreover, in this study, participants were asked for their perspective both in a direct way (description of criteria for good/bad consultations in general terms) and in a more indirect way (elaboration on concrete examples of good/bad consultations). By encouraging GPs to speak freely about concrete situations and analyzing the narratives given, this study aimed at gaining access to the reality that is constructed by the participants [17].

Presumably, the predominant use of specific discourses can in some cases be linked to external factors, such as work-related characteristics (e.g. work experience, practice characteristics) or accidental factors, (e.g. recent events, recent training). However, the data collected for this study do not permit an examination of possible correlations between discourses and external factors. Moreover, discourses are context specific [35]. In this study, only GPs working in the Flemish region of Belgium were recruited, which implies that all participants came from particular working conditions and medical training. Therefore, apart from being small, the sample used in this study was neither random nor representative (although attention was paid to obtain demographic variation in the sample). Concerning the methodology, the mere use of interview as data can be considered a limitation. Triangulation of the interview data with naturalistic data (e.g. written narrative material or actual doctor-patient interactions) could make the analysis more powerful. Moreover, further research on the implications of the variability in discourses used by GPs is needed.

Nevertheless, the outline of GPs’ discourses on clinical practice provided in this study can function as a framework to help GPs reflect on how they construct their own practice. This type of reflection is particularly relevant since variety in GPs’ discourses implies that a good match between doctor’s and patient’s perspectives is not self-evident. Rather than focusing on good doctor-patient fits, the GP’s ability to handle or to switch between different perspectives with regard to the same situation is considered useful. The framework that is presented in this study can also help GPs become more aware of their particular perception of medical practice, can help them manage the challenges met in daily practice and can enhance doctor-patient communication [36]. Participation in group discussions, such as Balint groups [37,38], where one is gently confronted with the limitations of the angle from which a situation is viewed, may also be helpful in this regard.

## Conclusion

This study clearly indicates that there is no uniform way in which GPs perceive clinical practice. Each of the participants used a subtle mix of different criteria to define good and bad medical consultations. Some discourse elements appear to be rooted in medical literature, whereas others are of a more personal nature. By focusing on the limitations of each discourse, this study can shed new light on some of the difficulties GPs encounter in their daily practice: being confronted with specific problems might be an effect of adhering to a specific discourse. The typification of different discourses on consultations may function as a framework to help GPs reflect on how they perceive their practice, and help them manage some of the challenges met in daily practice.

## Abbreviations

GP: General Practitioner.

## Competing interests

The authors declare that they have no competing interests.

## Authors’ contributions

KV conducted the interviews and made notes about observations and impressions during the interviews. KV and SV both coded the interview transcripts and discussed the codes as well as the emerging discourses. KV drafted the manuscript, which was extensively commented on by SV. MD brought along relevant literature, verified the final results and examined whether the discourses identified were supported by relevant interview fragments. All authors read and approved the final manuscript.

## Pre-publication history

The pre-publication history for this paper can be accessed here:

http://www.biomedcentral.com/1471-2296/14/62/prepub
